# Dual-Stimuli Responsive Carbon Nanotube Sponge-PDMS Amphibious Actuator

**DOI:** 10.3390/nano9121704

**Published:** 2019-11-28

**Authors:** Yu Ji, Yufeng Xing, Xuequan Li, Li-Hua Shao

**Affiliations:** Institute of Solid Mechanics, Beihang University (BUAA), Beijing 100083, China; jiyubuaa@buaa.edu.cn (Y.J.); xingyf@buaa.edu.cn (Y.X.); lixuequan@buaa.edu.cn (X.L.)

**Keywords:** dual-stimuli, actuator, CNT sponge

## Abstract

A dual-stimuli responsive soft actuator based on the three-dimensional (3D) porous carbon nanotube (CNT) sponge and its composite with polydimethylsiloxane (PDMS) was developed, which can realize both electrothermal and electrochemical actuation. The bimorph actuator exhibited a bending curvature of 0.32 cm^−1^·W^−1^ under electrothermal stimulation on land. The displacement of the electrochemical actuator could reach 4 mm under a 5 V applied voltage in liquid. The dual-responsive actuator has demonstrated the applications on multi-functional amphibious soft robots as a crawling robot like an inchworm, a gripper to grasp and transport the cargo and an underwater robot kicking a ball. Our study presents the versatility of the CNT sponge-based actuator, which can be used both on land and in water.

## 1. Introduction

Actuators, which can move in response to input energies [1,2,3], have had increasing attention paid to them over the years for different applications such as biomedical devices [4,5], soft robots [6,7] and artificial muscles [8,9]. Various smart materials have been utilized for actuation to meet different demands, such as the shape memory polymer (SMP) [10,11,12], shape memory alloy [13,14,15,16,17] shape memory hydrogels [18,19,20,21,22] and ionic electroactive polymer(including ionic polymer-metal composites, conductive polymer and polyelectrolyte [23,24,25]) to realize the flexibility, large actuation displacement and high actuation force properties [26,27,28,29]. Bodaghi et al. have designed adaptive structures of SMP to achieve self-expanding and self-shrinking by means of four-dimensional printing technology [12]. As the polymer-based actuator material, SMP usually experiences a large modulus change under temperature stimulus to accomplish actuation [10]. However, the high hardness of the SMP limits its application on soft actuators. Therefore, shape memory hydrogels have been investigated in recent years. Lendlein et al. have reported a shape memory hydrogel actuated by temperature change through crystallization and amorphization of hydrophobic side chains [30]. Chen et al. have fabricated a double layer self-deformed shape memory hydrogel which includes a thermo-responsive actuating layer and a pH-responsive memorizing layer [31]. Kaynak et al. have introduced 3D printed chitosan hydrogels soft actuators in liquid, which showed an electro-osmotic swelling response as a function of charge density and external salt concentration [25]. Compared with other stimuli such as heat, sunlight or pH, the electric stimulus takes advantages of convenience and accuracy. Therefore, the electrothermal actuators (ETA) and electrochemical actuators (ECA) both play important roles among various actuators. As the major component, carbon nanotube (CNT) [32,33,34,35] is commonly utilized in ETA or ECA owing to its exceptional properties, such as its light weight, high conductivity and large specific surface area. Fan et al. have reported the aligned CNT-based composite electrothermal actuator taking advantage of the mismatch in the coefficient of thermal expansion (CTE) of different components; the free-end displacement of the actuator could reach up to 9.5 mm in 5 s [32]. A twistable and bendable actuator with directional controllability, by designing the shape of the electrothermal bimetallic CNT-based PDMS composite actuator, has been fabricated [36]. Vaia et al. have also reported the reversible displacement of up to 2 mm of polyimide strips with CNT addition under a few hundred volts of applied voltage [37]. A review of ionic polymer metal composite (IPMC) actuators prepared with different kinds of CNT-based electrodes as ECA has been presented by Chen’s group [38]. As early as 1999, Baughman et al. firstly reported the Single-Wall Carbon Nanotubes based electrochemical actuator [39]. Then their group started to focus on the actuation performance of CNT based actuators. An all-solid-state torsional electrochemical yarn muscle has provided a tensile stroke of 1.3% and thermally powered CNT-hybrid yarn muscles filled with paraffin wax could generate tensile strokes of over 10% [40]. A coiled electrochemically driven CNT yarn artificial muscle has been fabricated that provided tensile strokes of up to 30 times higher than the previous electrochemical muscles [41]. CNT buckpapers have been used as electrodes with a Nafion membrane as the electrochemical actuation [42]. 

However, these ETAs or ECAs mentioned above only work either electrothermally or electrochemically, respectively. It will be highly desired if one actuator could be adaptive to different environments. Actuators based on a CNT/polymer bimorph that are responsive to light, humidity or electricity have been achieved [43]. Chen et al. have fabricated a dual-responsive actuator based on CNT-coated paper and biaxially oriented polypropylene (BOPP), which was driven by humidity and light [44]. Furthermore, a CNT sheet-based bimorph actuator driven by electricity, NIR light, humidity, and volatile organic vapors has also been developed [45]. An acidized single-walled CNT/low-density polyethylene with a thickness of less than 50 μm could be driven by multiple stimuli such as electricity, NIR light, and organic vapors [46]. Nonetheless, there is seldom research on a soft actuator functioning both in air and in liquid, which can be utilized as an amphibious robot. Lee et al. presented a high performance artificial muscle, which is based on the thermally or electrochemically induced volume changes of twist-spun CNT yarns [40]. In their work, the torsional and tensile actuations have been investigated. However, no one has worked on the bendable actuation base on carbon-polymer soft materials, which function both on land and in water.

Here, we fabricated a dual-stimuli responsive carbon nanotube sponge (CNT sponge) based actuator by combining the electrothermal actuation and the electrochemical actuation in air and in liquid, respectively. The CNT sponge has the excellent properties of mechanical flexibility, electrical conductivity and thermal stability, and has potential applications on hard actuators based on CNT sponge/SPM [47], sensors [48], supercapacitors [49] and fuel cells [50]. In this work, the three-dimensional porous CNT sponge PDMS-based actuators were investigated, which could be driven both electrothermally and electrochemically. Compared to the expensive fabrication and low-dimensional limitations of CNT sheet structure, the CNT sponge was synthesized by facile chemical vapor deposition, making it easier for mass production. The thickness of the sponge could be tailored freely, which was also convenient for applications as both a bimorph actuator and a three-dimensional compressive actuator. The soft actuator presented here could be used for biomimetic actuation owing to its high flexibility. The CNT sponge PDMS-based actuator is light-weight, flexible and adaptable to different environments with large actuation strain, and could be used as an amphibious soft robot on land and in water.

## 2. Materials and Methods 

### 2.1. Synthesis of CNT Sponge 

The CNT sponge was synthesized by chemical vapor deposition (CVD) using ferrocene (Shanghai Aladdin Biochemical Technology Co., Ltd, Shanghai, China) as the catalyst precursor and 1,2-dichlorobenzene (Shanghai Aladdin Biochemical Technology Co., Ltd, Shanghai, China) as the carbon source. A mixture of ferrocene powders dissolved in dichlorobenzene (0.06 g/mL) was injected into a quartz tube located in the CVD furnace (Zhonghuan Furnace Corporation, Tianjin, China) by a syringe pump (LongerPump LSP01-1A, Shaoxing, China) at a feeding rate of 0.16 mL/min. The carrier gas was a mixture of Ar and H_2_, which was flowing at the rate of 2000 mL/min and 300 mL/min, respectively. The growth procedure took 45 min under the reaction temperature of 860 °C, when the sponge reached a thickness of ca. 0.86 mm. More details of the sponge synthesis procedure can be found in Ref. [51]. Finally, the sponge was peeled off the quartz substrate after CVD. 

### 2.2. Fabrication of the Bimorph Actuator 

**Figure 1:**
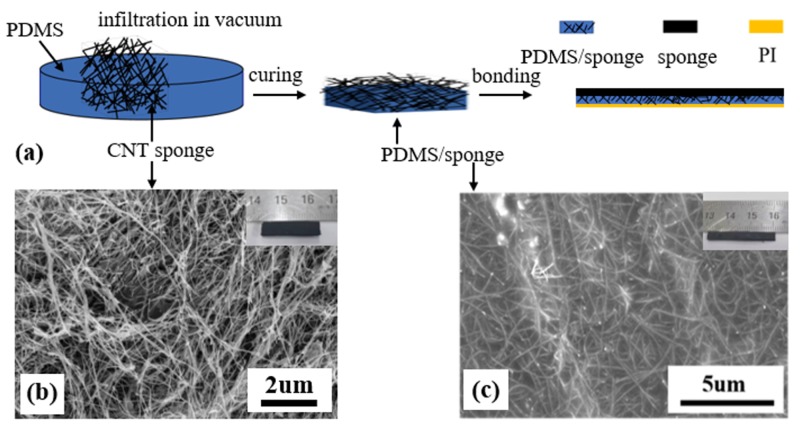
Fabrication and characterization of the actuator. (**a**) The schematic fabrication process of the CNT sponge/PDMS/PI actuator. (**b**) SEM image of a random stacking CNT network of sponge, and the inset is the macroscopic photo of the sponge. (**c**) SEM image of the sponge/PDMS composite, and the inset is the photo of the composite. Note that the porous CNTs are embedded in the PDMS matrix.

**Figure 2:**
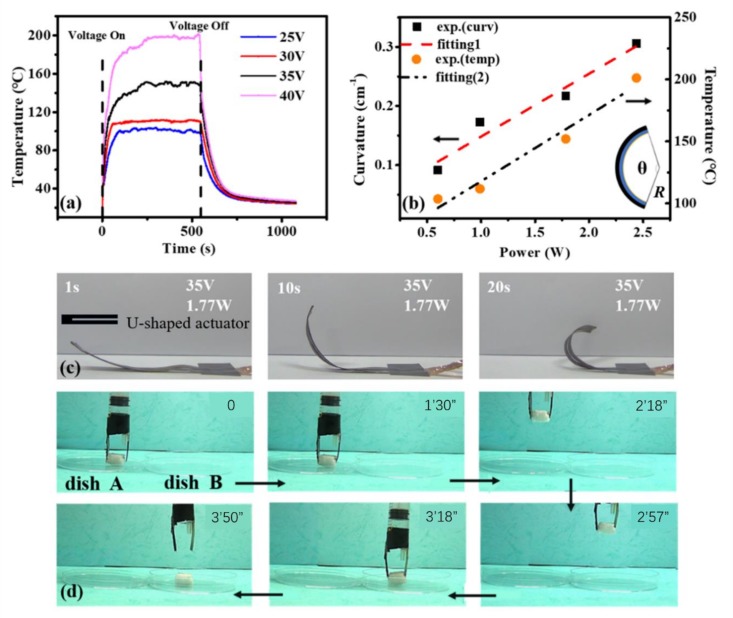
The electrothermal actuation performance of the CNT sponge/PDMS/PI bimorph actuator. (**a**) The temperature of the actuator change versus different applied voltages time. (**b**) The curvature (left axis) and temperature (right axis) of the actuator versus input power. The inset is the schematic plot of the geometry parameter. (**c**) Bending behavior of the bimorph actuator under 35 V voltage, and the inset is an illustration of the U-shaped actuator. (**d**) The demonstration of the gripper transferring a cargo under 25 V voltage.

In this work, the bimorph actuator consisted of three parts, which were the pure three-dimensional (3D) porous CNT sponge (abbreviated as sponge), sponge/PDMS composite and polymide (PI) tape. The fabrication method is schematically shown in Figure 1a. The synthesized CNT sponge was placed in a petri dish. Then an uncured PDMS solution (matrix mixed with a hardener at 10:1, SYLGARD 184, Midland, MI, USA) was poured in the dish until the thickness of ca. 0.65 mm was reached. We put the mixture in a vacuum-drying oven at room temperature for 1 h to allow the uncured PDMS solution to flow into the pores of the sponge and to remove the gas bubbles. Then, it was heated at 100 °C for 1 h to solidify the uncured solution. Lastly, PI tape was bonded to the composite of the CNT sponge/PDMS to prepare the bimorph actuator. Note that the composite (thickness of ~0.65 mm) was stuck on the PI tape and the other side was the unfilled pure CNT sponge (see Figure 1a). The PI tape was 12.5 μm in thickness. PI is a typical material with a small CTE (*α*_PI_ = 20 × 10^−6^·K^−1^), while PDMS has a large CTE of 310 × 10^−6^·K^−1^. The sponge/PDMS/PI sandwich strip can be cut into any desired shape. In this paper, the U-shaped actuator (see Figure 2a) was used for the electrothermal bimorph actuator with dimensions of ca. 42 mm × 5 mm × 0.77 mm, and the gripper with ca. 50 mm × 5 mm × 0.77 mm. The width of the slit in the U-shaped actuator was 1 mm. The strip actuators were used for the electrochemical bimorph actuator (ca. 50 mm × 1mm × 0.77 mm). The electrothermal bimorph actuator was controlled by DC power to generate heat. The actuation under electrochemical stimulation can be realized by the CNT sponge/PDMS composite directly without PI tape.

The electrochemical actuation of the as-prepared bimorph actuator was characterized through a three-electrode electrochemical system. We choose 2 M potassium chloride (KCl) aqueous solution as the electrolyte, Ag/AgCl in the same solution as the reference electrode and the Pt plate as the counter electrode. We have measured the electrochemical actuation with different concentrations of the solution, which are 0.5 M, 1 M and 1.5 M and 2 M aqueous KCl electrolyte, respectively. The actuation strain is larger with the higher concentration. Finally, 2 M KCl aqueous solution was used as the electrolyte, with which the actuator showed high strain and the concentration is not so high to avoid the oversaturation for long time operation. The alternating voltages were applied on the actuator for 2 s of 5 V and 0 V, respectively. 

The morphological and structural properties were investigated by a scanning electron microscope (SEM, S-4800, Tokyo, Japan). The characterization of electrochemical actuation was achieved by an electrochemical workstation (CHI660D, ChenHua, Shanghai, China). The bimorph actuator was driven by DC power supply (MS-605D, MaiSheng, Suzhou, China) directly. The temperatures of the bimorph actuator were measured by using a non-contact infrared camera (FLIR A655SC, Wilsonville, OH, USA).

## 3. Results

### 3.1. Morphology of the Sponge and Composite

The SEM images of the CNT sponge and the sponge/PDMS composite and their macroscopic pictures are shown in Figure 1b,c respectively. The as-grown random stacked CNTs form a sponge with nanofibers and pores as illustrated in Figure 1b. Unlike the aligned CNTs, the macroscopically three-dimensional CNT sponge has advantages of tunable three-dimensional size, low density and large specific surface area with the random distributed CNT network. After the infiltration of PDMS, the porous CNT sponge is embedded in the PDMS matrix to form the sponge/PDMS composite as shown in Figure 1c.

### 3.2. Electrothermal Actuation 

Figure 2a presents the time-dependent temperature profiles of the bimorph actuator with different applied voltages. The temperature of the actuator increases rapidly after the applied voltage is turned on until it reaches a steady-state temperature. When the voltage is off, the temperature drops slowly to room temperature. The saturation temperature increases with the increased applied voltage. Figure 2b illustrates that both of the curvature (left axis) and saturation temperature (right axis) of the actuator vary linearly versus the input power. The linear slopes of the curvature and temperature curves are 0.32 cm^−1^·W^−1^ and 63 °C·W^−1^, respectively. The actuator (with one end fixed) bends into an arc with the curvature radius, *R*, which can be measured from the photo of the actuator as schematically shown in the inset of Figure 2b. Therefore, the value of curvature *κ* = 1/*R* can be calculated. Figure 2c demonstrates the bending behavior of a U-shaped actuator under the applied voltage of 35 V. One end of the actuator is fixed while the other part is driven by the electrical stimulus. We take advantage of the electrothermal characteristics of the sponge/PDMS/PI actuator to achieve grasping and transportation functions as a gripper under the applied voltage of 25 V as illustrated in Figure 2d. The gripper is composed of two U-shaped actuators to facilitate the connection to the power supply. Two strips move closer as a result of the bending of the actuators under the applied voltage, then grasp the cargo and transport it to the destination. Thereafter, the voltage is switched off and the strips move apart to release the cargo. In such a way, the gripper can transport the cargo steadily.

Generally, the saturation temperature of the electrothermal actuator can be calculated by Equation (1) [52]:

(1) $$T_{s}=\frac{1}{cm}\left(\frac{V^{2}t}{R}-Q_{loss}\right)-T_{i}$$

where *V* and *R* are the applied voltage and the resistance, respectively. *m*, *c*, *T_s_*, *T_i_*, *t* and $Q_{loss}$ are the mass, specific heat capacity, saturation temperature, initial temperature, real time and the total heat loss, respectively. The saturation temperature can be reached when the input energy and $Q_{loss}\;$reach a dynamic balance. According to Equation (1), improving the response speed can be achieved by increasing the input voltage and decreasing the heat loss in the environment. As shown in Figure 2a, the temperature change is faster with the higher applied voltage, thus the higher response speed of the actuator. One can take advantage of Equation (1) to design the electrothermal actuator. 

The bending behavior of the bimorph actuator is basically caused by the asymmetrical stresses in the top and the bottom layers. In the electrothermal mode, the thermal stresses of the composite layer and the PI layer are caused by the large difference in CTE. With the temperature increasing in the actuator, the composite layer expands more than the PI layer. However, because of the solid bonding between the layers, the constraint force of the PI layer will lead to compressive stress which results in the bending of the bimorph actuator. 

**Figure 3:**
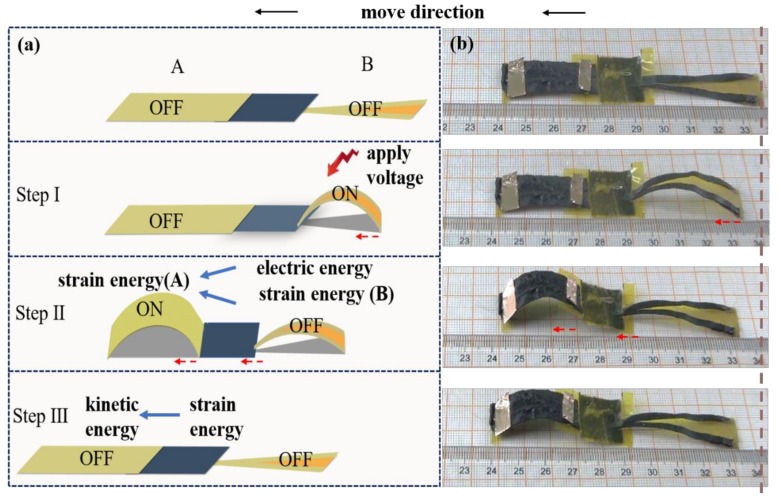
(**a**) Schematic illustration of the crawling motion generated by the robot. The robot contains two parts: the front body (A) and the legs (B). It takes three steps to crawl for one locomotion. Step I: apply 35 V voltage on B; Step II: apply 35 V voltage on A and turn off the power to B; Step III: turn off the power to A. (**b**) Snapshots of the wormlike robot crawling. Red arrow shows the moving direction. Step I: B moves forward; Step II: B recovers to straight state and A bends upward; Step III: part A moves forward.

An application as a crawling robot inspired by the inchworm movement is illustrated in Figure 3. The robot consists of two parts: the front body (A) and the legs (B). The movement includes alternate bending, recovering and locomotion of two parts of the robot, which can be achieved by three steps as shown in Figure 3. Step I: Firstly, applying 35 V voltage on B, the ends of the legs of B move forward due to the bending of B. Therefore, the electric energy transfers into the strain energy of B. Step II: Then applying 35 V voltage on A, the end of part A starts to move forward. Simultaneously, turn off the power to B, which leads to the recovering of B to the straight state. At the end of Step II, both the strain energy of B and the electric energy applied on A transfer into the strain energy of A. Step III, turning off the power applied to A, the front of A starts to move forward due to the recovering of A. The whole locomotion cycle takes ca. 60 s under 35 V applied voltage. The robot in Figure 3 has moved forward ca. 70 mm in 15 min. It will move faster if we use a higher voltage.

### 3.3. Electrochemical Actuation 

**Figure 4:**
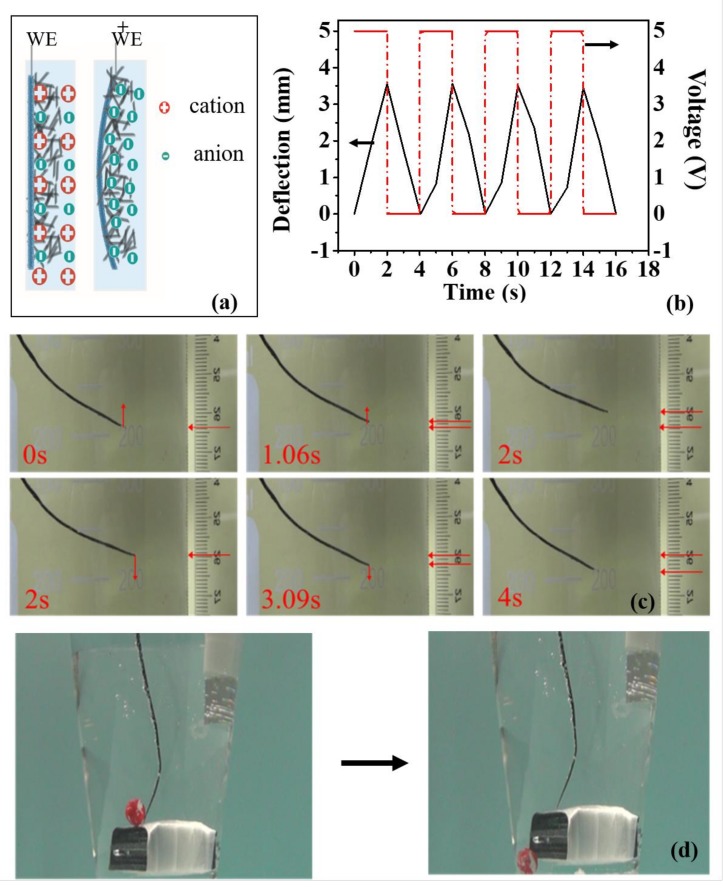
Characterization of electrochemical actuation of the bimorph actuator, when a 0.5 Hz square-wave voltage of 5 V was applied in 2 M potassium chloride solution. (**a**) Schematic diagram of the electrochemical actuation of the bimorph actuator. The CNT sponge/composite film as the working electrode, Ag/AgCl as the reference electrode and Pt plate as counter electrode. (**b**) The time dependence of applied potential (V, right axis) and resulting displacement (left axis) of the actuator. (**c**) Experimental photos of the actuation process. The displacement can reach up to 4 mm. (**d**) The actuator can be used as underwater robot kicking a ball.

The electrochemical bimorph actuator consists of the pure CNT sponge layer and the composite layer. The CNT sponge layer tends to expand or contract in response to the surface-stress change induced by charge injection, when the bimorph actuator is immersed in the electrolyte upon the variation of the applied voltage as schematically shown in Figure 4a. In contrast, the composite layer tends to maintain its original state. This will result in asymmetrical stresses between the two layers, and thus the bending of the actuator. The PI tape is unnecessary for the electrochemical actuator. Figure 4b shows the voltage dependence of the actuator displacement. When a 0.5 Hz square-wave voltage of 5 V is applied, the end of the bimorph actuator moves up to 4 mm. After the voltage decreases to 0 V, the actuator recovers to its initial state. When the actuator is positively charged, the insertion of anions would result in the contraction of the sponge layer. This behavior is consistent with that of a nanoporous carbon aerogel actuator, which is also a macroscopically three-dimensional material [53]. Meanwhile, the composite layer tends to maintain its original shape. Therefore, the asymmetrical stress results in the bending of the actuator. At the same time, bubbles can be observed on the actuator because of the electrolysis of water under this voltage (Figure 4c). These electrochemically induced gas bubbles on the surface of the carbon pores of the CNT sponge also contribute to actuation. This electrochemical actuator can function as an underwater robot. Figure 4d illustrates the robot kicking a red ball under the applied 1 Hz square-wave voltage of 5 V. 

The electrochemical-induced actuation of the bilayer structure with a porous layer and PDMS layer can be described by Equation (2) [54]. Assuming the strain of the porous layer caused by charge injection is *ε_p_*, the bilayer film will bend because of the difference in strain between the top and the bottom surfaces of the strip induced by the charge injection. The strain of the porous layer can be calculated by Equation (2):

(2) $$\varepsilon_{p}=\frac{1}{R}\frac{E_{p}^{2}h_{p}^{4}{\left(1-\upsilon_{s}\right)}^{2}+E_{s}^{2}h_{s}^{4}{\left(1-\upsilon_{p}\right)}^{2}+2E_{p}E_{s}h_{p}h_{s}\left(2h_{p}^{2}+2h_{s}^{2}+3h_{p}h_{s}\right)\left(1-\upsilon_{p}\right)\left(1-\upsilon_{s}\right)}{6E_{p}E_{s}h_{p}h_{s}\left(h_{p}+h_{s}\right)\left(1-\upsilon_{p}\right)\left(1-\upsilon_{s}\right)}$$

where *p* and *s* indicate parameters for the porous and solid layers, respectively; *E* is Young’s modulus; *ν* is Poisson’s ratio; *h* is the layer thickness; and *R* is the radius of curvature of the film. More detailed derivation can be seen in Ref. [54]. If the porous-layer strain remains unchanged, the smaller $h_{s}/h_{p}$, will lead to the smaller radius of curvature. Therefore, one can increase the thickness of the sponge to increase the displacement of the underwater robot.

In fact, we can control the thickness of the CNT sponge by changing the growth time of the CVD progress. Therefore, the actuator based on the CNT sponge can be fabricated not only in the shape of a sheet, but also a three-dimensional cube to bear the compression load. Only the bimorph film actuator is discussed in this paper, which can be used in soft robots amphibiously.

## 4. Conclusions

In this work, the 3D CNT sponge was synthesized and partially infiltrated within PDMS to fabricate the CNT sponge/PDMS based actuator. Both the bending curvature and temperature of the electrothermal actuator vary linearly versus the input power with linear slopes of 0.32 cm^−1^·W^−1^ and 63 °C·W^−1^, respectively. The displacement of the electrochemical actuator is up to 4 mm, when a 0.5 Hz square-wave voltage of 5 V is applied. The different applications of the amphibious soft robot have been demonstrated as a crawling robot, a gripper and an underwater robot for locomotion, transportation and kicking, respectively. The actuator based on the CNT sponge can be fabricated not only in the shape of a sheet, but also a three-dimensional cube to bear the compression load. In future work, the versatile actuators will be developed for more applications.
